# Utility of osteoporosis screening based on estimation of bone mineral density using bidirectional chest radiographs with deep learning models

**DOI:** 10.3389/fmed.2025.1499670

**Published:** 2025-03-26

**Authors:** Akifumi Yoshida, Yoichi Sato, Chiharu Kai, Yuta Hirono, Ikumi Sato, Satoshi Kasai

**Affiliations:** ^1^Department of Radiological Technology, Faculty of Medical Technology, Niigata University of Health and Welfare, Niigata. Japan; ^2^Nagoya University Graduate School of Medicine, Aichi, Japan; ^3^Major in Health and Welfare, Graduate School of Niigata University of Health and Welfare, Niigata, Japan; ^4^TOITU Co., Ltd., Tokyo, Japan; ^5^Department of Nursing, Faculty of Nursing, Niigata University of Health and Welfare, Niigata, Japan

**Keywords:** bone mineral density, osteoporosis, screening, chest radiograph, artificial intelligence

## Abstract

**Introduction:**

Osteoporosis increases the risk of fragility fractures, especially of the lumbar spine and femur. As fractures affect life expectancy, it is crucial to detect the early stages of osteoporosis. Dual X-ray absorptiometry (DXA) is the gold standard for bone mineral density (BMD) measurement and the diagnosis of osteoporosis; however, its low screening usage is problematic. The accurate estimation of BMD using chest radiographs (CXR) could expand screening opportunities. This study aimed to indicate the clinical utility of osteoporosis screening using deep-learning-based estimation of BMD using bidirectional CXRs.

**Methods:**

This study included 1,624 patients aged ≥ 20 years who underwent DXA and bidirectional (frontal and lateral) chest radiography at a medical facility. A dataset was created using BMD and bidirectional CXR images. Inception-ResNet-V2-based models were trained using three CXR input types (frontal, lateral, and bidirectional). We compared and evaluated the BMD estimation performances of the models with different input information.

**Results:**

In the comparison of models, the model with bidirectional CXR showed the highest accuracy. The correlation coefficients between the model estimates and DXA measurements were 0.766 and 0.683 for the lumbar spine and femoral BMD, respectively. Osteoporosis detection based on bidirectional CXR showed higher sensitivity and specificity than the models with single-view CXR input, especially for osteoporosis based on T-score ≤ –2.5, with 92.8% sensitivity at 50.0% specificity.

**Discussion:**

These results suggest that bidirectional CXR contributes to improved accuracy of BMD estimation and osteoporosis screening compared with single-view CXR. This study proposes a new approach for early detection of osteoporosis using a deep learning model with frontal and lateral CXR inputs. BMD estimation using bidirectional CXR showed improved detection performance for low bone mass and osteoporosis, and has the potential to be used as a clinical decision criterion. The proposed method shows potential for more appropriate screening decisions, suggesting its usefulness in clinical practice.

## 1 Introduction

Osteoporosis is a systemic bone disease that causes bone fragility due to loss of bone mass (1). Osteoporosis is the most significant risk factor for fragility fractures and has a substantial impact on life prognosis (1–3). Among these, lumbar spine and femur fractures significantly worsen quality of life (4). In addition, osteoporosis typically does not cause symptoms until a fracture occurs (5, 6). Delayed diagnosis of osteoporosis leads to fragility fractures, with an estimated 37 million fragility fractures occurring annually in people aged 55 years and older worldwide between 1990 and 2019 (7). Therefore, early detection of osteoporosis before its progression is essential to prevent fragility fractures (8, 9).

Cost-effective screening is required to reduce the incidence of fragility fractures caused by osteoporosis (10). Osteoporosis is examined by measuring bone mineral density (BMD). Dual-energy X-ray absorptiometry (DXA) is the most accurate method for measuring BMD and remains the gold standard for screening osteoporosis (11, 12). Individual BMD measurements are assessed based on differences and ratios against the young adult mean (YAM) in skeletally healthy young adults. The T-score is an assessment index widely used as an international standard, and is the difference in an individual’s BMD measurement against the YAM divided by the standard deviation of young adults’ BMD. Corresponding to the World Health Organization (WHO) diagnostic categories, –2.5 ≥ T-score indicates osteoporosis, and –1 > T-score > –2.5 indicates low bone mass (13). In some countries, the relative ratio of an individual’s BMD to YAM is also used; for example, the diagnostic criteria for osteoporosis and osteopenia in Japan define osteoporosis as a BMD less than 70% of the YAM (14). Thus, osteoporosis can be objectively diagnosed based on BMD measurements. However, the high cost of DXA equipment limits the availability of medical facilities that can perform DXA, and the low uptake of DXA measurements poses a challenge for the early detection of osteoporosis (13).

Effective screening opportunities should be expanded for the early detection of osteoporosis. It would be useful if BMD could be obtained from popular medical data other than bone mineral density testing, the risk of osteoporosis could be assessed, or medical examinations could be encouraged based on objective data. Previous studies have shown that the values obtained by analyzing anatomical features, such as the cortical thickness of the clavicle, spine, and ribs on radiographs, correlate with BMD and bone mass (15–17). Furthermore, many recent studies have used deep learning and other artificial intelligence (AI) methods to analyze medical images and obtain information for purposes other than the original use. Previous studies have used deep learning models to estimate BMD using radiographic images of the lumbar spine and hip joint as inputs (18, 19) and to identify the presence of osteoporosis (20, 21), indicating the possibility of identifying osteoporosis with high accuracy. However, because lumbar and hip radiography are performed mainly as adjuncts to orthopedic consultations, the target population for the method of osteoporosis screening using lumbar and hip radiographs is limited to orthopedic patients. It has been reported that ≥ 75% of all individuals for whom DXA testing is recommended do not undergo it (22–27). Thus, it remains important to promote screening more broadly to target individuals without orthopedic consultation or those who have not undergone DXA testing. A method that utilizes medical data obtained at a high frequency regardless of orthopedic symptoms could effectively extend osteoporosis screening opportunities.

Chest radiography is performed consistently in primary care and during health checkups and is the most widely and frequently performed basic diagnostic imaging technique worldwide. It would be promising to expand screening without requiring additional imaging if chest radiographs (CXRs) could be used to accurately assess the risk (28). Studies investigating approaches to detect osteoporosis from CXR have been reported, including studies using deep learning models that directly classified the prevalence of osteoporosis from CXR (29) and estimated BMD from CXR with patient information (30, 31). This BMD estimation approach would be more valuable for flexible use in clinical practice because BMD estimates could be used to identify osteoporosis and/or to consider the necessity for DXA testing. Sato et al. reported the detection of osteopenia with a cutoff T-score of –1.0, based on estimated BMD, and obtained a sensitivity of 90.14% (30). Meanwhile, the detection sensitivity for osteoporosis with a cutoff T-score of –2.5 was relatively low at 77.27%. A study evaluating the recommendation criteria for osteoporosis screening described a sensitivity of > 90% as necessary (32); for example, the National Osteoporosis Foundation guideline criteria in the United States were evaluated and found to yield a sensitivity of 96.2% and specificity of 17.8% for selecting cases with T-score ≤ –2.5 in postmenopausal women aged ≥ 45 years (32). Improving the sensitivity of osteoporosis detection is essential to promote adequate osteoporosis screening opportunities and to provide more persuasive recommendations for medical examinations in recipients undergoing chest radiography.

In primary care and health checkups in many countries, chest radiography with frontal and lateral bidirectional imaging is often performed depending on the patient’s symptoms (33). A lateral CXR can visualize the thoracic region up to the upper lumbar spine, and the shape of the vertebrae can be observed. Imaging findings of fragility fractures such as vertebral compression fractures are also diagnostic criteria for osteoporosis (13), and lateral CXRs are effective for detecting vertebral fractures (34). Previous studies using lateral CXR have been reported on osteoporosis screening using lateral CXR with automatic detection using AI (35). Previous studies using AI to estimate BMD from medical images tended to show higher estimation performance when using radiographs in which the region to be measured by DXA was imaged than when it was not (36). Therefore, we hypothesized that AI with input of lateral CXR can estimate BMD effectively, and that combining frontal and lateral CXR images will improve the accuracy of AI estimation compared with using only the frontal image as input.

This study aimed to develop deep learning models to estimate BMD using only bidirectional CXR as input and to clarify their performance improvement over models using only frontal CXR as input, as well as their utility in osteoporosis screening.

## 2 Materials and methods

### 2.1 Materials

#### 2.1.1 Data

This study was approved by the institutional review boards of the participating institutions (approval number: 18973-230111). The Institutional Review Board determined that formal informed consent was not required because this study used de-identified clinical data.

**FIGURE 1:**
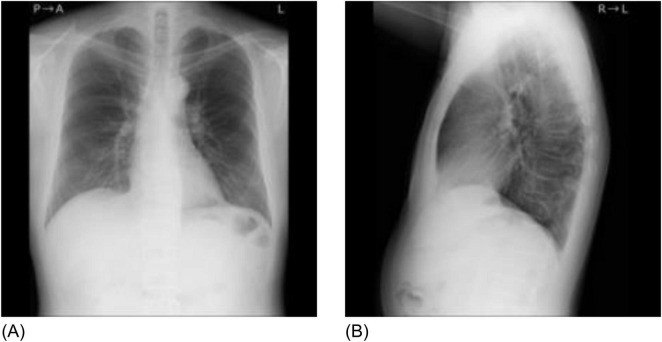
Sample chest radiographs of one patient used in this study. **(A)** Frontal chest radiograph, **(B)** Lateral chest radiograph. The original images were down-sampled and zero-padded to 1,024 × 1,024 matrixes with the aspect ratio preserved and resized to 299 × 299 as the input size for the pre-trained model.

This study included patients aged ≥ 20 years who underwent bidirectional CXR and BMD measurement using DXA within 1 year at a single medical facility in Japan from April 2010 to July 2022. This study included DXA examinations performed at the facility during the study period and corresponding chest radiographs (frontal and lateral) performed at the facility within 1 year before and after the DXA examination. All the paired data were incorporated within the inclusion period. Data were excluded if the CXR lacked parts of the lungs or clavicles and if the CXR was performed using portable devices. A total of 1,624 cases (520 males, 1,104 females) with 6,446 data pairs (2,682 males, 3,764 females) of multiple BMD measurements and bidirectional CXR images met these criteria. The data attributes are listed in Table 1. A Horizon (Hologic Inc., Marlborough, MA, United States) was used for BMD measurements. Lumbar BMD measured in the lumbar vertebral region and femoral BMD measured in the femoral neck region were targeted. T-scores and BMD/YAM were calculated based on the mean BMD values and standard deviations for each sex of young adults, corresponding to the DXA device used for actual BMD measurements. According to the WHO (Geneva) criteria, the participants were classified based on their T-scores into normal (T-score ≥ –1.0), osteopenia (–1.0 < T-score < –2.5, and osteoporosis (T-score ≤ –2.5) groups (37). All bidirectional CXRs used in this study were obtained using frontal and lateral chest radiography. Figure 1 shows an example of a bidirectional CXR. Each original CXR image of different sizes was resized while preserving the aspect ratio and was zero-padded to a 1,024 × 1,024 matrix.

**TABLE 1:** Summary of data characteristics.

**Demographics**		
**Sex,** *n* (%)		
Male	2,682	(41.6%)
Female	3,764	(58.4%)
**Age,** *mean* [*IQR*] (years)	60.0	[47, 75]
**BMI,** *mean* [*IQR*] (kg/m^2^)	20.3	[17.7, 22.2]
**Bone mineral density**		
**Lumbar BMD**, *mean* [*IQR*] (g/cm^2^)	0.893	[0.770, 1.012]
**Femoral BMD**, *mean* [*IQR*] (g/cm^2^)	0.616	[0.516, 0.711]
**T-score,** *n* (%)		
T-score ≥ –1.0	1,373	(21.3%)
–2.5 < T-score < –1.0	2,411	(37.4%)
T-score ≤ –2.5	2,662	(41.3%)
**BMD/YAM,** *n* (%)		
BMD/YAM ≥ 80%	2,650	(41.1%)
70% < BMD/YAM < 80%	1,373	(21.3%)
BMD/YAM ≤ 70%	2,423	(37.6%)

Age, BMI, lumbar BMD, and femoral BMD are shown as mean [25, 75 percentiles]. *n* is the number of applicable data-pairs and not the number of participants. BMI, body mass index; BMD, bone mineral density; IQR, Interquartile range; YAM, young adult mean.

#### 2.1.2 Dataset splitting

A 10-fold cross-validation method was used to create the datasets; each of the 10 datasets was created by randomly splitting the participants into training, validation, and evaluation ratios of 80%, 10%, and 10%, respectively. In each dataset used for cross validation, there was no contamination by the same participants between the training, validation, and evaluation data. The AI outputs for the ten evaluation datasets were combined, and all data were used for evaluation.

#### 2.1.3 Experimental environment

The specifications of the experimental computer were an Intel Core i5-12400 CPU, 16 GB × 2 of RAM, and an NVIDIA GeForce RTX 3090 GPU with a VRAM of 24 GB. The experiment was implemented using MATLAB 2023b (MathWorks, Inc.) on Windows 11 (Microsoft, Inc.). All image processing and deep learning network computations were performed using MATLAB.

### 2.2 Methods

#### 2.2.1 Experiments

**FIGURE 2:**
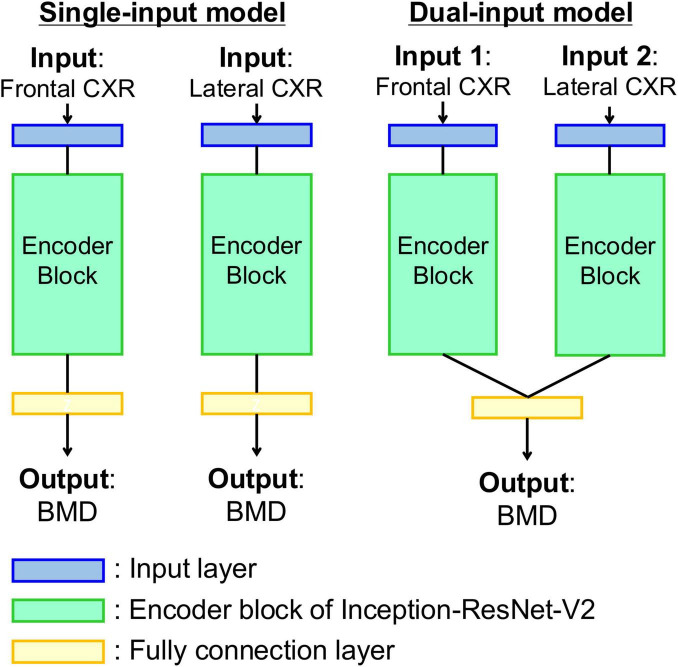
Diagrams of the model for estimating bone density from chest radiographs. **(Left)** Single-input model with input of frontal or lateral chest radiograph. **(Right)** Dual-input model with inputs of bidirectional chest radiographs. Each encoder block is composed of a pre-trained Inception-ResNet-V2 network as feature extractor.

The Inception-ResNet-V2 model (38) was used as the feature extractor. The model data were acquired from the public MATLAB add-on library (39). The model data were used as a pretrained network obtained from a classification task using ImageNet (40). The classifier in the base model was replaced by a single fully connected layer, with a single output class as the regression layer. The models used in the experiments were a single-input model, which used one direction of the CXR as the input, and a dual-input model, which used a bidirectional CXR as the input. Figure 2 shows a structural diagram of the models. Using the single-input model, training was conducted to estimate the BMD using the CXR frontal or lateral views as the input. The dual-input model was implemented using two single-input models trained on frontal and lateral CXR. The trained feature extractors of the single-input models were connected in parallel to the fully connected layer. The dual-input model was trained to estimate the BMD using paired frontal and lateral CXRs inputs. The training conditions were as follows: optimization method Adam, loss function root mean square error, learning rate 1 × 10^–3^, 3 × 10^–4^, 1 × 10^–4^, 3 × 10^–5^, 1 × 10^–5^ (variable), batch size 32, maximum number of epochs 100, and image data augmentation with ± 5° rotation, horizontal flipping, and ± 5% scaling. The learning rate was selected to obtain the lowest loss-function value for each model. The input images were resized to the input size of the pretrained model (299 × 299 pixels) during image data augmentation and then used as the model input. The training dataset was used to update the network weights and the validation dataset was used to display the model performance at each epoch. Training was stopped early when the loss value for the validation dataset at the end of each epoch did not update the minimum for 10 consecutive epochs. The weights at the epoch with the smallest loss function output on the validation dataset were saved, thereby completing the training. Model training was conducted separately for each target to be estimated as the lumbar and femoral BMD.

#### 2.2.2 Evaluation

The trained model was used to input chest X-ray images from the evaluation dataset and output BMD estimates. The Pearson’s correlation coefficient (*R*) and mean absolute percentage error (MAPE) between the reference measured values and model estimates were used as the evaluation metrics. A 95% confidence interval (CI) of the correlation coefficient was obtained using Fisher’s z-transform. A Bland–Altman analysis was performed using the mean values of the reference measured values and model estimates and the differences between the estimates and measured values. If the following conditions were met, the estimated values and measured values were considered equivalent: (1) The 95% CI of the mean difference between the estimated and measured values included ± 0.01 g/cm^2^, and (2) more than 95% of the evaluation data were included within the limits of agreement (LOA) of mean ± 1.96 SD (41).

The predicted T-score and BMD/YAM ratio were calculated using the predicted BMD and actual YAM according to sex. Based on the WHO guidelines, osteoporosis (measured T-score ≤ –2.5) and osteopenia (measured T-score < –1.0) were detected from the calculated predicted T-scores. Additionally, the detection performance for osteoporosis and osteopenia was evaluated. Furthermore, based on Japanese guidelines (13), measured BMD/YAM ≤ 70% (osteoporosis) and < 80% (low bone mass) were detected from the predicted BMD/YAM. The evaluation metrics included sensitivity, specificity, positive predictive value (PPV), negative predictive value (NPV), and the area under the receiver operating characteristic curve (AUROC).

## 3 Results

The results of the BMD estimation for each model with different inputs are presented in Table 2. The model with bidirectional CXR inputs showed superior correlation coefficients to models with only frontal or lateral image inputs: *R* = 0.766 (95% CI 0.756–0.776) and 0.683 (95% CI 0.670–0.696) for lumbar and femoral BMD estimation, respectively.

**TABLE 2:** Prediction results for bone mineral density of the lumbar spine and femoral neck by deep learning models with three different input type.

	*R* [95% CI]		MAPE [95% CI]/%	
**Lumbar BMD**				
Frontal	0.709	[0.682–0.707]	11.5	[11.3–11.8]
Lateral	0.732	[0.720–0.743]	11.0	[10.8–11.3]
Bidirectional	**0.766**	[0.756–0.776]	**10.6**	[10.4–10.9]
**Femoral BMD**				
Frontal	0.581	[0.565–0.597]	14.7	[14.3–15.1]
Lateral	0.624	[0.609–0.638]	14.7	[14.3–15.0]
Bidirectional	**0.683**	[0.670–0.696]	**13.8**	[13.5–14.1]

BMD, bone mineral density; CI, confidence interval; *R*, Pearson’s correlation coefficient; MAPE, mean absolute percentage error. Bolded values of R and MAPE represent the best value for each BMD.

**FIGURE 3:**
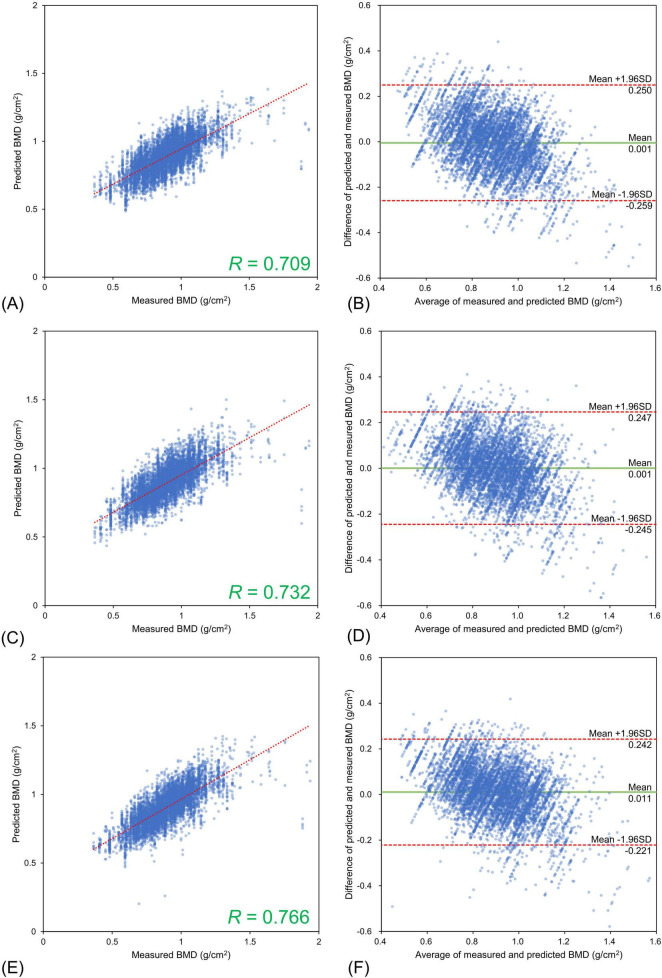
Estimation results for bone mineral density of the lumbar spine using three different input types. Upper **(A,B)** Frontal chest radiographs (CXR) input, Middle **(C,D)** Lateral CXR input, Bottom **(E,F)** Bidirectional CXR input. Left **(A,C,E)** Relationships of measured and estimated values, Right **(B,D,F)** Bland–Altman plots. All the models confirmed the agreement between model estimates and dual-energy absorptiometry measurements.

**FIGURE 4:**
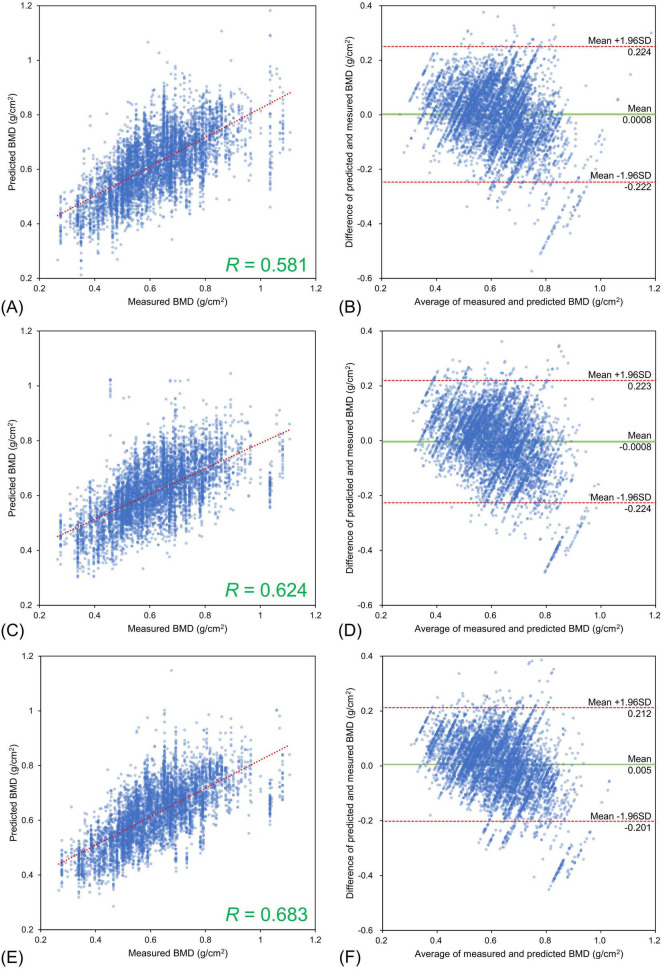
Estimation results for bone mineral density of the femoral neck using three different input types. Upper **(A,B)** Frontal chest radiographs (CXR) input, Middle **(C,D)** Lateral CXR input, Bottom **(E,F)** Bidirectional CXR input. Left **(A,C,E)** Relationships of measured and estimated values, Right **(B,D,F)** Bland–Altman plots. All the models confirmed the agreement between model estimates and dual-energy absorptiometry measurements.

The lumbar BMD estimation results for each input image type relative to the DXA measurements are shown in Figure 3 and the femoral BMD estimation results are shown in Figure 4. The statistics for the Bland–Altman analysis are shown in Table 3. In the Bland–Altman analysis based on lumbar BMD estimates by the three models and actual measurements by DXA, the 95% CIs for the mean of the differences between the estimates and measurements overlapped with the reference range, indicating no fixed errors. The percentage of data within the LOA was greater than 95% for all three models. These results confirm the agreement between the lumbar BMD estimates and measured values in the three models with different input types. Bland–Altman analysis for femoral BMD estimation showed that the 95% CIs of the mean differences between the model estimates and the DXA measurements overlapped with the reference range, indicating no fixed errors. The percentage of data within the LOA was greater than 95% for all three models. These results confirm the agreement between the femoral BMD estimates and actual measurements for the three models with different input types.

**TABLE 3:** Summary of statistics in Bland–Altman analysis.

			Limits of agreement	
	**Mean difference [95% CI] (g/cm^2^)**		**Range (g/cm^2^)**	**Agreement/%**
**Lumbar BMD**				
Frontal	–0.0011	[–0.0079 to –0.0016]	–0.262 to 0.250	96.1
Lateral	0.0009	[–0.0021 to 0.0040]	–0.245 to 0.247	95.6
Bidirectional	0.0106	[0.0077 to 0.0135]	–0.221 to 0.242	95.5
**Femoral BMD**				
Frontal	0.0017	[–0.0014 to 0.0048]	–0.247 to 0.250	97.2
Lateral	–0.0033	[–0.0061 to –0.0005]	–0.226 to 0.220	95.6
Bidirectional	0.0052	[0.0026 to 0.0078]	–0.202 to 0.212	95.7

CI, confidence interval; BMD, bone mineral density.

**FIGURE 5:**
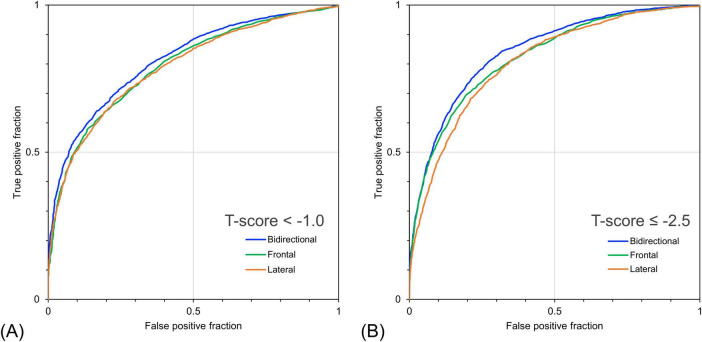
Receiver operating characteristic curves for detecting bone loss and osteoporosis based on bone mineral density (BMD) T-score using predicted bone mineral density. **(A)** Detection performance for cases with measured T-score < –1.0 and **(B)** ≤ –2.5. The bidirectional chest radiographs (CXR) input model performed better than the single-view models, particularly for detecting cases with a T-score ≤ –2.5.

Table 4 shows the detection performance for low bone mass and osteoporosis using the estimated T-score obtained by the models with different inputs. The model with the bidirectional CXR input yielded the highest values for all other indicators, except for sensitivity. In the model with bidirectional CXR inputs, the detection performance for T-score < –1.0 and T-score ≤ –2.5 were 90.0% and 63.4% for sensitivity, 46.0% and 85.6% for specificity, and 81.5% and 84.2% for AUROC, respectively. The model with bidirectional CXR inputs improved specificity and AUROC by approximately 2.2% and 2.1% for T-score < –1.0 and 4.2% and 1.9% for T-score ≤ –2.5, respectively, compared with the model with frontal CXR input. Figure 5 shows the ROC curves for detecting low bone mass (measured T-score < –1.0) and osteoporosis (measured T-score ≤ –2.5) using predicted T-scores. For osteoporosis detection, the model with bidirectional CXRs showed better performance than those with only frontal or lateral CXRs.

**TABLE 4:** Detection results with predicted values by different models for osteoporosis and osteopenia based on T-score of measured bone mineral density.

	Accuracy	Sensitivity	Specificity	PPV	NPV	AUROC/%
**BMD T-score < –1.0**						
Frontal	79.5	89.1	43.8	85.4	52.1	79.4%
Lateral	78.9	**90.3**	36.9	84.1	50.7	79.0%
Bidirectional	**80.6**	90.0	**46.0**	**86.0**	**55.4**	**81.5**%
**BMD T-score ≤ –2.5**						
Frontal	74.5	**64.7**	81.4	71.0	76.6	82.3%
Lateral	72.6	62.1	79.9	68.5	75.0	80.7%
Bidirectional	**76.5**	63.4	**85.6**	**75.6**	**76.9**	**84.2**%

Accuracy, Sensitivity, Specificity, PPV, NPV, and AUROC are shown as percentage values. BMD, bone mineral density; PPV, positive predictive value; NPV, negative predictive value; AUROC, area under the receiver operating characteristic curve. Bolded values represent the best in each BMD T-score category.

**FIGURE 6:**
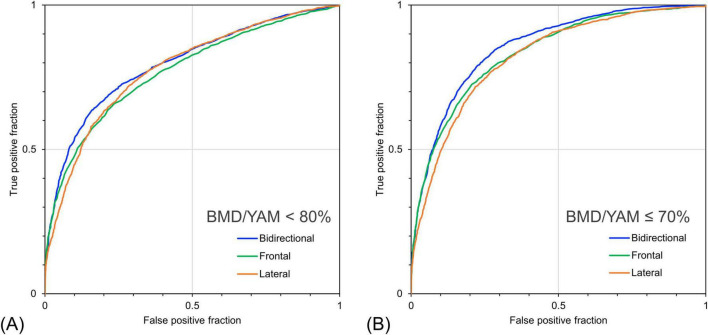
Receiver operating characteristic curves for detecting bone loss and osteoporosis based on the ratio of individual bone mineral density to the young adult mean (BMD/YAM) using predicted bone mineral density. **(A)** Detection performance for cases with measured BMD/YAM < 80% and **(B)** ≤ 70%. The bidirectional chest radiographs (CXR) input model performed better than the single-view models, particularly for detecting cases with BMD/YAM ≤ 70%.

Table 5 shows the detection performance for low bone mass and osteoporosis based on the estimated BMD/YAM derived from the models with different inputs. The model with bidirectional CXR yielded the highest values for almost all indices except for sensitivity. The model with bidirectional CXR improved the specificity and AUROC for detecting osteopenia with a BMD/YAM cutoff of 80% by 5.3% and 2.5%, respectively, and for detecting osteoporosis with a BMD/YAM cutoff of 70% by 3.0% and 2.2%, respectively, compared with the model with only frontal CXR input. Figure 6 shows the ROC curves for the detection of low bone mass and osteoporosis using the predicted BMD/YAM ratios. The model with bidirectional CXR showed higher performance for osteoporosis detection than those with only frontal or lateral images.

**TABLE 5:** Detection results with predicted values by different models for osteoporosis and osteopenia based on the ratio of measured bone mineral density to the young adult mean.

	Accuracy	Sensitivity	Specificity	PPV	NPV	AUROC/%
**BMD/YAM < 80%**%						
Frontal	69.4	75.3	61.0	73.5	63.3	77.2%
Lateral	71.0	**79.1**	59.5	73.6	66.5	78.0%
Bidirectional	**71.6**	75.2	**66.3**	**76.2**	**65.1**	**79.7**%
**BMD/YAM ≤ 70%**						
Frontal	76.2	**61.5**	85.0	71.2	78.6	83.4%
Lateral	74.9	58.7	84.6	69.6	77.3	82.0%
Bidirectional	**77.8**	60.8	**88.0**	**75.4**	**78.9**	**85.6**%

Accuracy, Sensitivity, Specificity, PPV, NPV, and AUROC are shown as percentage values. PPV, positive predictive value; NPV, negative predictive value; AUROC, area under the receiver operating characteristic curve; BMD, bone mineral density; YAM, the young adult mean. Bolded values represent the best in each BMD/YAM category.

## 4 Discussion

The results of this study suggest that ensemble learning models with frontal and lateral CXR inputs yield a higher BMD estimation accuracy than models with single-view CXR inputs. To the best of our knowledge, this is the first study to develop a deep learning model to estimate BMD using only lateral and frontal CXR images. The results of the ensemble model with bidirectional CXR inputs indicated higher accuracy of osteoporosis screening in clinical practice. Furthermore, our findings add value to the previous studies on BMD estimation using chest radiographs as an input. The lateral CXR images contained information related to BMD, and the effectiveness of the lateral CXR images for estimating BMD using AI was demonstrated.

The results of lumbar spine BMD estimation using bidirectional CXR (frontal and lateral) showed a strong positive correlation of *R* = 0.766 with the actual DXA measurements, indicating the feasibility of obtaining a stronger correlation than that obtained in previous studies. Among previous studies on BMD estimation using chest radiographs, Sato et al. reported the highest estimation performance (30). Using CXR frontal images and patient information (age and sex) as model inputs, they obtained *R* = 0.68 for lumbar BMD estimation and *R* = 0.75 for femoral BMD estimation. Meanwhile, the estimation of femoral BMD using bidirectional CXRs in this study showed a moderate correlation of *R* = 0.683, which was relatively weak. The average of 5,157 training data points in the 10-fold cross-validation in this study was about 58.8% less than the 12,529 training data points used by Sato et al. Generally, the performance of deep learning models increases with the number of training data points. Our model with bidirectional CXR inputs has the potential to achieve even higher estimation performance with greater amounts of training data.

The developed dual-input model with bidirectional CXR inputs is more effective than the single-input model in terms of application to osteoporosis screening. The estimates from the model with bidirectional CXR inputs in this study identified the presence of a measured T-score of < –1.0, with 90% sensitivity and 46% specificity, and a cutoff predicted T-score of –1.0. Regarding triage screening for osteoporosis, a sensitivity of > 90% and a specificity of approximately 40%–60% or higher are considered acceptable clinical decision criteria (32), and our results with a fixed cutoff fulfilled these criteria for the identification of a T-score < –1.0. Furthermore, suppose that the cut-off of the predicted T-score based on the model output is tuned according to this criterion. In such cases, it can provide even higher sensitivity for the detection of osteoporosis. For the detection task for cases with T-score ≤ –2.5, the cutoff of predicted BMD was modified to 40% and 50% specificity. Table 6 lists the sensitivities of the models with different input information when the specificity was tuned. The model with bidirectional CXR inputs showed a sensitivity of 94.5% at a specificity of 40% and a sensitivity of 91.3% at a specificity of 50% for detecting a measured T-score ≤ –2.5. Thus, the developed model may be helpful for screening for osteoporosis and low bone mass. The sensitivity of the model with bidirectional CXR as input at 50% specificity was 91.3% for T-score ≤ –2.5, which was + 2.5% higher than that of the model with frontal CXR as input. The model with only frontal CXR showed a sensitivity of 88.8%, which was less than acceptable for the clinical decision criteria. These results indicate the usefulness of deep learning methods using bidirectional CXR for osteoporosis screening.

**TABLE 6:** Sensitivities of the models with different inputs in detecting risk groups for osteoporosis based on measured BMD, with variable cutoffs based on model estimates and tuned specificity.

	Sensitivity/%	
	**With 40% specificity**	**With 50% specificity**
**BMD T-score ≤ –2.5**		
Frontal	**93.5**	88.8
Lateral	**92.4**	89.1
Bidirectional	**94.5**	**91.3**
**BMD T-score < –1.0**		
Frontal	**90.1**	86.1
Lateral	89.7	85.3
Bidirectional	**92.0**	88.4
**BMD/YAM ≤ 70%**		
Frontal	**94.9**	**90.7**
Lateral	**93.7**	**91.0**
Bidirectional	**95.8**	**92.8**

Bolded values represent sensitivity above 90%, Underlined values are the highest values for the three input types. BMD, bone mineral density; YAM, the young adult mean.

The results of the estimates from the bidirectional CXR model showed the feasibility of effective screening for osteoporosis and osteopenia based on the T-score and BMD/YAM criteria. A T-score ≤ –2.5 is a current global diagnostic criterion for osteoporosis. However, other criteria have been used to diagnose osteoporosis in other regions. For example, the Japanese criteria are based on BMD/YAM (37). Future diagnostic criteria may change based on the medical conditions. In addition, the management of osteoporosis risk groups is essential. Therefore, it is important to provide a robust detection performance for diagnoses based on various cutoff values. As shown in Table 6, the results of this study with bidirectional CXRs met the sensitivity > 90% and specificity > 40% levels for multiple cutoffs of T-score ≤ –2.5, T-score < –1.0, and BMD/YAM ≤ 70%. Thus, BMD prediction using bidirectional CXRs can provide robust screening performance for osteoporosis and its risk groups based on multiple cut-off values.

This study used only CXR images as model input to estimate BMD and did not use patient information such as age, sex, or clinical covariates. Previous studies estimating BMD from CXR used a model with frontal CXR and patient information (30, 31), although it was not clarified to what extent the AI model performance was affected by the input of patient information. The reason patient information was not input in this study was to eliminate the influence of the input of patient information on the estimation results since the purpose was to clarify the differences in performance depending on the input of CXR imaging direction and number of inputs. Another study reported that the use of patient information such as age, sex, and body mass index improved model performance (42). In future studies, our model may improve the accuracy of osteoporosis screening in primary care and health check-ups by inputting patient information and clinical covariates. This study aimed to propose an expansion of opportunity for osteoporosis screening by estimating BMD from chest radiographs, which are frequently obtained in primary care. Regarding opportunistic screening using chest radiographs, studies have reported on the estimation of pulmonary function (43–45) and the prediction of cardiovascular events (46) using deep learning models. These studies have proposed the secondary use of chest radiographs acquired for other purposes to incidentally detect the risk of lifestyle-related diseases. This study could be applied concurrently with these approaches to provide an opportunity to intervene before disease progression.

This study had two limitations. Firstly, this study used data obtained from a single facility to train and evaluate the model, which may have been affected by the regional characteristics. To validate the generalizability of this study’s findings, it is important to evaluate their performance using data collected from different facilities and/or racial groups. Secondly, the diagnostic results of chest radiography were not considered. The results of this study could potentially have been influenced by imaging findings; however, their influence is unclear.

## 5 Conclusion

This study developed a deep learning model to estimate BMD using frontal and lateral CXRs and demonstrated the utility of the model for osteoporosis screening based on the model estimates. The model with bidirectional CXR inputs showed higher BMD estimation performance than the model with a single CXR input. This suggests the usefulness of a BMD estimation model using bidirectional CXR inputs for screening for osteoporosis and low bone mass.

## Data Availability

The raw data supporting the conclusions of this article will be made available by the authors, without undue reservation.
